# Metagenome-Assembled Genome Sequences of Three Uncultured *Planktomarina* sp. Strains from the Northeast Atlantic Ocean

**DOI:** 10.1128/MRA.00127-20

**Published:** 2020-03-19

**Authors:** Matilde Marques, Nuno Borges, Sandra Godinho Silva, Ulisses Nunes da Rocha, Asunción Lago-Lestón, Tina Keller-Costa, Rodrigo Costa

**Affiliations:** a iBB–Institute for Bioengineering and Biosciences, Department of Bioengineering, Instituto Superior Técnico, Universidade de Lisboa, Lisbon, Portugal; b Helmholtz Centre for Environmental Research (UFZ), Leipzig, Germany; c Department of Medical Innovation, Centro de Investigación Científica y de Educación Superior de Ensenada (CICESE), Ensenada, Mexico; d Centro de Ciências do Mar (CCMAR), Universidade do Algarve, Faro, Portugal; e Department of Energy Joint Genome Institute, Berkeley, California, USA; f Lawrence Berkeley National Laboratory, Berkeley, California, USA; Georgia Institute of Technology

## Abstract

We report three metagenome-assembled genomes (MAGs) of *Planktomarina* strains from coastal seawater (Portugal) to help illuminate the functions of understudied *Rhodobacteraceae* bacteria in marine environments. The MAGs encode proteins involved in aerobic anoxygenic photosynthesis and a versatile carbohydrate metabolism, strengthening the role of *Planktomarina* species in oceanic carbon cycling.

## ANNOUNCEMENT

Bacterioplankton communities are key players in nutrient cycling in the world’s oceans (1). The *Roseobacter* clade-affiliated (RCA) cluster (*Rhodobacteraceae*, *Alphaproteobacteria*) has been found to constitute up to 35% of coastal marine bacterioplankton, to be widely distributed from temperate to polar waters, and to play an important role in the degradation of phytoplankton-derived organic matter (1–5). The genus *Planktomarina* is a dominant member of the RCA community (3, 6), with the photoheterotrophic type strain Planktomarina temperata RCA23 isolated in 2013 from the Wadden Sea (2). We report three metagenome-assembled *Planktomarina* sp. genomes from the Northeast Atlantic Ocean that represent a novel candidate species within the RCA cluster.

Three seawater samples were obtained at ca. 18-m depth off the coast of Faro, Algarve, Portugal (latitude 36.979778, longitude –7.989111) in June 2014. Samples were filtered through sterile 0.22-μm nitrocellulose membrane filters, and the total community DNA was extracted using the UltraClean soil DNA isolation kit (Mo Bio Laboratories) (7; T. Keller-Costa, A. Lago-Leston, J. P. Saraiva, R. Toscan, S. G. Silva, J. Gonçalves, C. J. Cox, N. C. Kyrpides, U. Nunes da Rocha, and R. Costa, submitted for publication). DNA libraries were prepared using the Nextera DNA sample preparation kit from Illumina and subjected to paired-end metagenome sequencing (Illumina HiSeq 2500; depth, ∼20 million 101-bp reads per sample) with 200 cycles. The raw sequence reads were trimmed and quality checked with the Trim Galore module with MetaWRAP v1.0.5 (8). Assemblies were performed with the quality-filtered, unmerged reads using metaSPAdes (9). Eukaryotic contigs, assigned with EukRep (10), were thereafter filtered out. The metagenome-assembled genomes (MAGs) were then binned from the obtained prokaryotic-enriched assemblies with MetaBAT2 (11) and subjected to taxonomic classification with GTDB-Tk v0.3.2 (12) on the KBase server (13) and with the Microbial Genomes Atlas (MiGA) (14). Three MAGs identified as a *Planktomarina* sp. (strains SW01_Bin08, SW02_Bin14, and SW04_Bin04) showing standard completeness and contamination scores were chosen for this study (Table 1). The average nucleotide identity blast (ANIb) and average amino acid identity (AAI) values were calculated between MAGs and *P. temperata* RCA23 using JSpeciesWS (15) and aai.rb (16), respectively. Functional annotations were performed with the RAST server (17), and clusters of orthologous groups (COGs) of proteins and protein families (Pfams) were predicted with WebMGA (18). antiSMASH v5.0 (19) was used to identify biosynthetic gene clusters. Default parameters were used for all software unless otherwise noted.

**TABLE 1:** General features of metagenome-assembled *Planktomarina* genomes from the Northeast Atlantic Ocean

Isolate	Original sample^*a*^ (BioSample no.)	Compl/cont^*b*^	Size (bp)	No. of contigs	No. of CDSs^*c*^	*N*_50_ (bp)	GC content (%)	ANI (%)^*d*^	AAI (%)^*d*^	Genome accession no.
SW04_Bin04	SW04 (SAMEA3913377)	78.4/1.8	2,419,173	144	2,489	27,162	54.8	87.43	91.94	GCA_902732755
SW01_Bin08	SW01 (SAMEA3913374)	72.1/1.8	2,208,500	136	2,255	22,913	55.0	87.56	92.19	GCA_902732765
SW02_Bin14	SW02 (SAMEA3913375)	80.2/2.7	2,269,649	125	2,326	27,294	55.0	87.46	91.95	GCA_902732775

a The original metagenome BioSample from which the MAG was obtained.

b Compl, completeness score; Cont, contamination score.

c CDSs, coding DNA sequences.

d ANI and AAI were obtained with MiGA against the genome sequence of type strain *P. temperata* RCA23 (accession number GCA_000738435).

The three MAGs shared 99% ANI and AAI among themselves but presented only 87.5% ANI with *P. temperata* RCA23 (Table 1), suggesting that they may represent a novel *Planktomarina* species. Nevertheless, the MAGs shared 1,217 “core” COGs with *P. temperata* RCA23 out of 1,575 “pangenome” COGs detected across all four genomes. Among the common features between the MAGs and the *P. temperata* RCA23 genome, we highlight genes encoding proteins involved in aerobic anoxygenic photosynthesis (e.g., chlorophyll *a* synthase, EC 2.5.1.62) and several metallo-beta-lactamases associated with antibiotic resistance, along with a terpenoid gene cluster showing 100% similarity to a carotenoid cluster (BGC0000647) from Rhodobacter sphaeroides. Strengthening the role of *Planktomarina* spp. in carbon cycling, multiple glycoside hydrolase genes (e.g., alpha-amylase [PF00128], alpha-galactosidase [EC 3.2.1.22], and beta-galactosidase [EC 3.2.1.23]) were also common to all genomes, with chitinase-encoding genes (EC 3.2.1.14) being furthermore detected in the *P. temperata* RCA23 and SW01_Bin08 genomes.

### Data availability.

The metagenome-assembled genomes as well as the raw metagenome data have been deposited at ENA/EMBL/GenBank under the study accession number PRJEB13222. The BioSample accession numbers of the MAGs are SAMEA6497282 through SAMEA6497284, and their GenBank accession numbers are GCA_902732755 (SW04_Bin04), GCA_902732765 (SW01_Bin08), and GCA_902732775 (SW02_Bin14). The MAGs were obtained from the marine metagenome BioSamples SW01 (SAMEA3913374), SW02 (SAMEA3913375), and SW04 (SAMEA3913377).
